# The continued citation of retracted publications in dentistry

**DOI:** 10.5195/jmla.2020.824

**Published:** 2020-07-01

**Authors:** Nicole R. Theis-Mahon, Caitlin J. Bakker

**Affiliations:** 1 theis025@umn.edu, Health Sciences Libraries, University of Minnesota, Minneapolis, MN; 2 cjbakker@umn.edu, Bio-Medical Library, University of Minnesota, Minneapolis, MN

## Abstract

**Objective:**

Publications are retracted for many reasons, but the continued use and citation of retracted publications presents a problem for future research. This study investigated retractions in the dental literature to understand the characteristics of retracted publications, the reasons for their retractions, and the nature and context of their citations after retraction.

**Methods:**

In September 2018, the authors identified retracted dentistry publications using the Retraction Watch database. Citations to those publications were retrieved from Scopus and Web of Science. Characteristics of retracted publications and their citations were collected, including study design, reasons for retraction, and nature of citation (positive, negative, or neutral). We used chi-square tests to determine if there were notable differences between retracted publications that were cited following retraction and those that were not, and if there were relationships between the nature of the citation, the study design of the original publication, and its reason for retraction.

**Results:**

Of the 136 retracted publications, 84 were cited after retraction. When restricted to English language, 81 retracted publications received citations from 685 publications. Only 5.4% of the citations noted the retracted status of the original publication, while 25.3% of citations were neutral and 69.3% were positive. Animal studies were more likely to be uncited after retraction, while in vitro studies and randomized controlled trials were more likely to be cited. Retracted publications that were cited negatively were more likely to have been retracted due to scientific distortion than those that were cited positively or neutrally. Retracted publications that were cited negatively were also more likely to be observational studies than those cited positively or neutrally.

**Conclusion:**

Retracted publications in dentistry are continually cited positively following their retraction, regardless of their study designs or reasons for retraction. This indicates that the continued citation of retracted publications in this field cannot be isolated to certain research methods or misconduct but is, instead, a more widespread issue.

## INTRODUCTION

The increasing prevalence of retracted publications in the literature presents a concern to research integrity. Although retracted publications represent a very small proportion of the scholarly literature—an estimated 0.02%—the annual number of retracted publications reported in MEDLINE grew 42% between 2013 and 2017 [1]. Because research builds on the work that comes before it, this growing number of retracted publications presents a potential problem to the continued advancement of scientific research.

Several reasons have been proposed to explain the growing number of retractions. An ever-increasing number of scholarly journals, increased rates of article publication, growing issues with the peer-review process, and challenges associated with university incentive systems worldwide have been put forward as potential influences in the rate of retractions [2–5]. The growth of retracted publications could also be attributed in part to increasing awareness of retractions and their potential impact, and broadening adherence to guidelines put forward by journal publishers and organizations such as the Committee on Publication Ethics [6–8]. The intention of such policies is to enhance the accountability, integrity, and transparency of scientific research [9].

Previous research has documented and analyzed retracted publications in a variety of biomedical disciplines, including drug therapy [10], radiation oncology [11], oncology [12], radiology [13], emergency medicine [14], surgery [15], and orthopedics [16]. In the dental literature, previous studies have found that retracted publications most frequently report on in vitro studies, observational studies, cases, and narrative reviews [17–19]. These studies provide a context for understanding the characteristics of the retracted publications; however, they do not explore the impact of retractions in dentistry. The scope and span of the dental literature is vast, varying from bench science, dental materials, and biomaterials to clinical research, community health, and health policy. This provides a unique landscape in which to explore the impact of retractions.

The continued citation of retracted publications has been a subject of concern for several decades [20–23]. Garfield acknowledged the diverse reasons work could be cited, from “paying homage to pioneers” and colleagues to disputing or criticizing previously published research [24]. Despite this initial recognition that citation is not inherently endorsement, others have since argued that “it is somewhat of an achievement to have one's work noticed by others, even if negatively; work deemed substandard or negligible is seldom cited at all” [25].

Guidelines and policies largely address circumstances under which publications should be retracted and offer general guidance on pragmatic aspects of retraction, including best practices for metadata and discoverability. Despite these policies, there are notable inconsistencies in whether and how retraction notices and the retracted statuses of publications are displayed across databases. In their study of the representation of retractions in mental health publications, Bakker and Riegelman have found that 40% of records for retracted publications did not indicate that the publication had been retracted [26].

To understand the potential impact of retractions, it is important to recognize that publications can be retracted for many reasons, ranging from publishing errors, such as articles being published in the wrong issue, to the widespread fabrication of data. These various reasons produce a range of potential consequences for science. Bar-Ilan and Halevi have described three categories for reasons for retraction: (1) administrative error, such as publishing errors; (2) ethical misconduct, such as plagiarism, duplicate publication, lack of institutional review board (IRB) approval, or interference with the peer-review process; and (3) scientific distortion, such as data falsification or data fabrication, or unsupported conclusions. In Bar-Ilan and Halevi's categorical classification schema, scientific distortion is considered to be the most problematic [2]. Scientific distortion, even when unintentional, nevertheless constitutes “hurdles for the advancement of science, as [articles] mislead scientists who rely on the results of such articles” [2]. Such work may redirect resources from other avenues of research based on misinformation. Administrative error, the third category, includes “other reasons for retraction that have no influence on the advancement of science” [2].

Coupling the reason for retraction and the nature in which retracted publications continue to be cited after retraction reveals the problems that they present by supporting future research and science. Bar-Ilan and Halevi have presented a theoretical framework in their contextual analysis of fifteen retracted publications: positive citations are those in which a publication is cited as if it had not been retracted and is used to support or corroborate the article's findings; neutral citations are those in which an article is cited “as a publication that appears in the literature and [the citing publication] does not include judgement of its validity”; and negative citations are those that note the retracted status of the publication being cited [27]. This framework assigns an overall value to the retracted publication, thus allowing researchers to understand the overall impact post-retraction.

The aim of this study was to investigate retractions in the dental literature in order to understand the overall characteristics of retracted publications, the reasons for their retraction, and the nature of their post-retraction use.

## METHODS

The authors identified a set of retracted publications in the dental literature from the Retraction Watch Database by downloading all citations in the Dentistry category that were available as of September 2018 [28]. This resource, which is developed and maintained by the Center for Scientific Integrity, is an expansion of their work with the Retraction Watch blog, which reports on retractions of scholarly publications and related topics.

We recorded study designs, as defined by the American Dental Association (ADA) Center for Evidence-Based Dentistry [29], for the list of retracted publications. Cohort, cross-sectional, and case control studies were collapsed into the group of “observational studies,” and in vitro and animal studies were broken out into separate categories. Guidelines were classified as “other.” The final publication types included in our analysis were animal study; case report; expert opinion (including narrative reviews); in vitro; observational (cohort, cross-sectional, case control); other; randomized control trial; and unknown (publication for which a study design could not be determined based on title and abstract, and for which full text was not available). We assigned a reason for retraction based on the theoretical framework developed by Bar-Ilan and Halevi that included administrative error, ethical misconduct, and scientific distortion [2].

In October 2018, we conducted known item searching in Scopus and Web of Science to identify citations to these retracted publications. Citations were reviewed, and citations to the retracted publication that occurred prior to its retraction notice were excluded. This was determined by date of article submission, where available. Citations to retraction notices were also removed.

We created a form in Qualtrics to record information about the citations. This included language, study design, location of the citation (e.g., introduction, methods, results, discussion), sentence or section citing the retraction, and the nature of the citation. The nature of the citation was defined in accordance with Bar-Ilan and Halevi's categorizations: positive, neutral, or negative [27]. For articles with split sentiments, in other words, multiple citations of differing natures, a single sentiment was assigned. For example, if the first citation was neutral and the second one was positive, the overall sentiment would be positive. Double data entry was conducted for all citing articles, and disagreements were discussed until consensus was reached.

Statistical tests were performed using R, version 3.6.0 [30]. Chi-square tests were conducted to examine relationships between study designs, reasons for retraction, likelihood of being cited post-retraction, and nature of subsequent citations (positive, negative, or neutral). Descriptive statistics regarding the retracted publications are also presented.

## RESULTS

The original set of 136 retracted articles, with a publication date range of 1998–2018, were cited 1,409 times. The majority were retracted due to ethical misconduct (n=80, 58.8%) and scientific distortion (n=37, 27.2%). The most prevalent study designs were observational studies (n=35, 25.7%), case reports (n=25, 18.4%), and in vitro studies (n=25, 18.4%). Of the 84 studies that were cited after retraction, ethical misconduct (n=53, 63.1%) and scientific distortion (n=22, 26.2%) were the leading reasons for retraction, and observational (n=25, 29.8%) and in vitro studies (n=19, 22.6%) were the most prevalent study designs. Details of the studies are described in Table 1 and Table 2.

**Table 1:** Retracted publication means and median

	All retracted publications (n=136)	Retracted publications cited after retraction (n=84)
Mean times cited	10.96	16.15
Median times cited	3.00	6.00
Mean time between publication and retraction	2.09 years	2.04 years

**Table 2:** Characteristics of retracted publications

	All retracted publications (n=136)		Retracted publications cited after retraction (n=84)	
	n	(%)	n	(%)
Reason for retraction				
Ethical misconduct	80	(58.8%)	53	(63.1%)
Scientific distortion	37	(27.2%)	22	(26.2%)
Unknown	9	(6.6%)	5	(6.0%)
Administrative error	10	(7.4%)	4	(4.8%)
Study design				
Observational	35	(25.7%)	25	(29.8%)
In vitro	25	(18.4%)	19	(22.6%)
Case report	25	(18.4%)	13	(15.5%)
Randomized clinical trials (RCTs)	15	(11.0%)	12	(14.3%)
Expert opinion	20	(14.7%)	11	(13.1%)
Animal study	12	(8.8%)	4	(4.8%)
Other	3	(2.2%)	0	(-)
Unknown	1	(0.7%)	0	(-)

The 136 retracted publications were considered in a binary manner (cited after retraction or not). The influence of reasons for retraction and study designs on whether the publication would be cited after retraction were analyzed using the chi-square test of independence (Table 3). A statistically significant relationship was found between study design and citation after retraction (χ^2^(7, 136)=17.607, *p*=0.014): animal studies were more likely to be uncited after retraction, whereas in vitro studies and randomized controlled trials were more likely to be cited after retraction.

**Table 3:** Comparison of retracted publications that are cited or uncited post-retraction

	Cited after retraction (n=84)		Not cited after retraction (n=52)		*p*
	n	(%)	n	(%)	
Reason for retraction					
Ethical misconduct	53	(63.1%)	27	(51.9%)	p=0.405
Scientific distortion	22	(26.2%)	15	(28.8%)	
Unknown	5	(6.0%)	4	(7.7%)	
Administrative error	4	(4.8%)	6	(11.5%)	
Study design					
Observational	25	(29.8%)	10	(19.2%)	p=0.014
In vitro	19	(22.6%)	6	(11.5%)	
Case report	13	(15.5%)	12	(23.1%)	
RCTs	12	(14.3%)	3	(5.8%)	
Expert opinion	11	(13.1%)	9	(17.3%)	
Animal study	4	(4.8%)	8	(15.4%)	
Other	0	(-)	3	(5.8%)	
Unknown	0	(-)	1	(1.9%)	

We excluded 3 articles that were not in English from the set of 84 publications that were cited after retraction, leaving 81 articles for further analysis. The publications were retracted between 2004 and 2018, with the average publication being retracted in 2009, 2.04 years after it had been published. The average citation occurred 4.1 years after the initial publication had been retracted. Positive citations on average occurred 4.2 years after retraction, neutral citations occurred 4.3 years after retraction, and negative citations occurred 4.0 years after retraction.

The 81 publications included in further analysis were cited in 685 publications. The majority (69.3%) of citations to retracted publications were positive, 25.3% were neutral, and 5.4% were negative. Chi-square tests found statistically significant relationships when considering the nature of citation and both the reason for retraction (χ^2^(6, 685)=24.703, *p*<0.0004, Table 4) and the study design of the retracted publication (χ^2^(10, 685)=42.036, *p*<0.0001, Table 5). Regarding the reasons for retraction, retracted publications that were cited negatively were more likely to have been retracted due to scientific distortion than those that were cited positively or neutrally. Regarding study design, retracted publications that were cited negatively were more likely to be observational studies than those that were cited positively or neutrally.

**Table 4:** Reason for retraction and the nature of citations

Nature of citation	Reason for retraction									*p*
	Administrative error (n=33)		Ethical misconduct (n=295)		Scientific distortion (n=337)		Unknown (n=20)		Total	
	n	(%)	n	(%)	n	(%)	n	(%)		
Positive	22	(4.6%)	199	(41.9%)	241	(50.7%)	13	(2.7%)	475	0.0004
Neutral	11	(6.4%)	89	(51.4%)	66	(38.2%)	7	(4.0%)	173	
Negative	0	(-)	7	(18.9%)	30	(81.1%)	0	(-)	37	

**Table 5:** Study design and the nature of citations

Nature of citation	Study design													*p*
	Animal study (n=28)		Case report (n=42)		Expert opinion (n=117)		In vitro (n=141)		Observational (n=259)		RCT (n=98)		Total	
	n	(%)	n	(%)	n	(%)	n	(%)	n	(%)	n	(%)		
Positive	22	(4.6%)	31	(6.5%)	78	(16.4%)	99	(20.8%)	179	(37.7%)	66	(13.9%)	475	<0.001
Neutral	4	(2.3%)	10	(5.8%)	39	(22.5%)	40	(23.1%)	50	(28.9%)	30	(17.4%)	173	
Negative	2	(5.4%)	1	(2.7%)	0	(-)	2	(5.4%)	30	(81.1%)	2	(5.4%)	37	

## DISCUSSION

To the best of our knowledge, this study was the first post-retraction analysis in the dental literature. Our findings illustrated the complexities that retracted publications present to the dental literature with their continued use and citation patterns. In our analysis, we could not identify significant factors that predicted the perpetuation of positive citations, including the reasons for retraction (scientific distortion, ethical misconduct, or administrative error) or the study designs of the retracted publications.

The initial 136 publications were most frequently retracted due to ethical misconduct (n=80, 58.8%) or scientific distortion (n=37, 27.2%), while only 7.4% of the sample were retracted due to administrative error. This result supported Faggion et al.'s findings that only 5.8% of articles in their sample were retracted due to publisher error, while 72.5% were retracted due to some form of misconduct, including plagiarism, overlap with other publications, inaccurate findings, or falsified results [17]. Reasons for retraction have varied in previously published literature, depending on elements such as the taxonomy that was employed [16, 31].

Certain study designs, specifically randomized controlled trials and in vitro studies, were more likely to be cited following retraction, while animal studies were less likely to be cited. This reflected previous research that highlighted citation patterns in all literature and indicated that randomized clinical trials (RCTs) were significantly more likely to be cited in comparison to animal studies [32]. One could argue that the statistical significance of study designs that we found in our study with regard to whether a paper was cited or not cited following retraction actually reflected citation patterns of the discipline overall, rather than being unique to retracted publications.

That statistically significant findings were largely associated with negative citations, rather than positive or neutral citations, was meaningful. This association might indicate that, regardless of the rationale for the retraction—whether the product of minor administrative error or widespread data fraud—there was no influence on the subsequent citation, use, and endorsement of the research. While we found that retracted publications that were cited negatively were more likely to have been retracted due to scientific distortion than those that were cited positively or neutrally, it should be noted that twenty-seven of these citing studies referenced the publications of a single researcher. This researcher, Jon Sudbø, acknowledged that the data underlying his publications were entirely fabricated, and the investigation into this misconduct generated interest among a broad range of researchers and clinicians [33–36]. The notoriety of this case likely contributed to the citation of these publications, and their representation in this data set created the potential for skewing the results. The lack of contribution to statistical significance of positive or neutral overall sentiment with study design or reason for retraction—or the overall rate of positive, negative, and neutral citation—reinforces the need for caution when considering these results.

The nature of the citations to the retracted articles in our study was predominately positive and neutral. Very few citations were negative, which reflected findings from other studies [17, 37]. The overall nature of the citations to retracted publications were overwhelmingly positive, regardless of the study designs of the retracted publications or the reasons for retraction. This perpetuation of positive citation was particularly problematic considering Bar-Ilan and Halevi's taxonomy of the reasons for retraction, which positioned scientific distortion as the most detrimental to scientific progress, followed by ethical misconduct. In our sample, these reasons for retraction accounted for 86% of all retracted publications. This percentage was slightly higher than similar studies that have researched retractions in the cancer and surgical literature [9]. Given that 37 of the 136 publications were retracted for scientific distortion—22 of which were cited following retraction—it is worrisome to consider how such citations may have influenced and potentially misdirected scientific progress due to fraudulent or invalidated research.

This leads one to question why researchers continue to use and cite retracted publications in their research. A variety of reasons may contribute to the perpetuation of retracted publications in the dental literature. These include, but are not limited to, inconsistencies in the display of retraction notices in databases and inconsistencies in the adoption of reporting guidelines in the dental literature [6, 38, 39]. Information-seeking behaviors also vary between dental researchers, who are more likely to have access to online resources from an academic institution, compared to those in private practice, as evidenced by a recent systematic review that found that those in clinical practice preferred print materials [40]. This reliance on a static copy could partially explain the perpetuation of retracted publications in the dental literature since researchers may be unaware of the retraction, thus perpetuating its use.

It should also be noted that the publication process can be long, with estimates of review times for articles being between 100 and 150 days [41, 42], which does not account for the substantial time spent developing the manuscript. The time between when the researchers initially conduct a literature search to when they submit that article for publication could include the period when the cited article was retracted. The responsibilities of the submitting authors, journals, and peer reviewers in ensuring the quality of underlying research articles has not been clearly articulated. This may be especially problematic in cases where the article in question is caught in cycles of review and revision. While we attempted to account for this by limiting our included citations to those that were submitted following the publication of the retraction notice, we recognize that authors may simply be unaware of the retracted status of the publication.

While some retracted publications, or portions of them, may have value for future research, their continued use poses a problem for future studies [25]. In evaluating retracted materials and their retraction notices, it is not always possible to determine which aspects of the retracted studies were valid or questionable. This creates challenges when attempting to determine whether specific data points or conclusions are sound, despite the retracted status of the publication. Greater clarity in retraction notices and their effective association with the original article could help to alleviate this difficulty by highlighting the questionable aspects of the underlying research.

Librarians are well positioned to support researchers in better understanding the complexity and potential impact of retracted publications. They have long assisted researchers in accessing a range of information sources and developing skills to critically appraise and synthesize information, and their “success in fulfilling those needs depends on the available literature, and having reliable and accurate information is critical” [43]. Libraries are also increasingly engaging in publishing services and providing guidance on optimizing research work flows and ensuring the reproducibility of research [44, 45]. Librarians can raise awareness regarding this complicated landscape, while advocating for improved practices among researchers and journals.

### Limitations

Retracted publications in this study were limited to those included in the Dentistry category of the Retraction Watch database. While this is not a comprehensive list of retracted publications in the dental literature, it represents a substantial sample that is reflective of the publication patterns in the field. The sample size in some of our categories was also small, particularly when considering certain study designs and the reasons for retraction. A small number of non-English language studies were also excluded from our analysis because of the subjective nature of determining the positive, neutral, or negative nature of the citation. As a result, our findings might not be aligned with those in non-English language dental literature. We recognize the subjective nature in general in determining the positive, neutral, or negative nature of citations. We attempted to account for this subjectivity by employing a rigorous screening process that involved a calibration exercise with a sample set and an independent screening process by both authors at every stage of the selection and data extraction process. Finally, we recognize that the assignation of a single sentiment where multiple citations and sentiments exist does eliminate some of the nuance from our analysis.

## CONCLUSION

Retracted publications continue to have an impact in the dental literature even after their retraction notices have been posted. In this study, 81 previously retracted publications received citations from 685 publications. The majority of these citations were positive or neutral, meaning the retracted publications were considered valid research or no judgment was offered. While we found associations between being cited negatively and the original publication's study design and reason for retraction, these results are likely due to the overrepresentation of retracted publications about a single researcher. Advances need to be made to clearly and uniformly identify retracted publications throughout the submission, review, and revision of manuscripts to ensure that retracted publications are not positively cited in future dental literature.
